# Three models for the regulation of polygenic scores in reproduction

**DOI:** 10.1136/medethics-2020-106588

**Published:** 2021-01-18

**Authors:** Sarah Munday, Julian Savulescu

**Affiliations:** 1 Medicine, Nursing and Health Sciences, Monash University, Clayton, Victoria, Australia; 2 Oxford Uehiro Centre for Practical Ethics, Faculty of Philosophy, University of Oxford, Oxford, UK; 3 Biomedical Ethics Research Group, Murdoch Children's Research Institute, Melbourne, Victoria, Australia; 4 Melbourne Law School, University of Melbourne, Melbourne, Victoria, Australia

**Keywords:** genetic selection, genetic counselling/prenatal diagnosis, regulation, mentally disabled persons, future child disability

## Abstract

The past few years have brought significant breakthroughs in understanding human genetics. This knowledge has been used to develop ‘polygenic scores’ (or ‘polygenic risk scores’) which provide probabilistic information about the development of polygenic conditions such as diabetes or schizophrenia. They are already being used in reproduction to select for embryos at lower risk of developing disease. Currently, the use of polygenic scores for embryo selection is subject to existing regulations concerning embryo testing and selection. Existing regulatory approaches include ‘disease-based' models which limit embryo selection to avoiding disease characteristics (employed in various formats in Australia, the UK, Italy, Switzerland and France, among others), and 'laissez-faire' or 'libertarian' models, under which embryo testing and selection remain unregulated (as in the USA). We introduce a novel 'Welfarist Model' which limits embryo selection according to the impact of the predicted trait on well-being. We compare the strengths and weaknesses of each model as a way of regulating polygenic scores. Polygenic scores create the potential for existing embryo selection technologies to be used to select for a wider range of predicted genetically influenced characteristics including continuous traits. Indeed, polygenic scores exist to predict future intelligence, and there have been suggestions that they will be used to make predictions within the normal range in the USA in embryo selection. We examine how these three models would apply to the prediction of non-disease traits such as intelligence. The genetics of intelligence remains controversial both scientifically and ethically. This paper does not attempt to resolve these issues. However, as with many biomedical advances, an effective regulatory regime must be in place as soon as the technology is available. If there is no regulation in place, then the market effectively decides ethical issues.

## Preimplantation genetic diagnosis and reproduction

In the late 1990s,^1^ the development of preimplantation genetic diagnosis (or PGD) made it possible to test in vitro fertilised (IVF) embryos for known genetic diseases and select only unaffected embryos for implantation. Some 20 years later, it is now commonplace for IVF embryos to be tested for monogenic disorders and/or chromosomal disorders such as Down syndrome.^1^ Moreover, testing is also available during non-IVF pregnancies.

Emerging technology promises further transformational change. Polygenic risk scores or polygenic scores (PS) analyse an individual’s genome, aggregating thousands of genes, to estimate genetic tendency towards particular traits and diseases.^2^ When PS are created from a large number of individuals, these can be plotted on a bell curve, to give results as a (more interpretable) percentile ranking. This process requires knowing which genes are relevant for the trait of interest, and which variants of these genes will promote or reduce its expression.

### The potential power of PS

According to the WHO, in 2016 four of the top five leading causes of death worldwide (and 9 of the top 10 in high-income countries) were non-communicable diseases (ischaemic heart disease, stroke, chronic obstructive pulmonary disease and dementia) with risk determined by both genetic and environmental factors.^3^ These diseases are typically polygenic, with a number of genes combining to confer different levels of risk on individuals, in combination with environmental factors. PS can be used to estimate the risk of developing each of these conditions, and, just as with monogenic disorders, IVF parents could select an embryo with lowest risk.

So how powerful are these tests? The development of PS requires studying genetics and human traits in hundreds of thousands—or even millions—of people. This has only become possible in recent years, with the development of large human genetics databases. These tests are limited: they can only be used to estimate genetic contributions to developing a condition. The heritability of each trait will provide the ultimate ceiling of predictive ability.

Nevertheless, for some diseases, current predictive power is increasing. Most studies evaluating the power of PS to detect disease risk use the ‘area under the curve’ (AUC) as a statistical test of efficacy. The AUC is a test which ‘describes the inherent validity of diagnostic tests’.^4^ It is presented as a value between 0.5 and 1.0, with 1.0 representing a perfect test, and 0.5 a test which is no better than chance. If a particular test has an AUC of 0.72, there is a 72% chance that it will be able to correctly identify the subject with disease when applied to select between one afflicted and one healthy participant.^4^ Generally, scores above 0.9 are deemed ‘outstanding’, 0.8–0.9 is ‘excellent’ and 0.7–0.8 is ‘acceptable’.^5^

One disease for which PS are providing promising results is type 1 diabetes mellitus (T1DM). According to a 2020 systematic review, the current most powerful PS for T1DM has an AUC of 0.92.^6^ When detecting early-onset T1DM, this value was increased to 0.96.^7^ A genomic prediction-led study applied a T1DM PS to select between siblings in families with a known history of T1DM. When compared with random selection, the score generated a relative risk reduction (of selecting an affected individual) ranging from 45% (when selecting between two siblings) to 72% (when selecting between five siblings).^8^ This approach mirrors the use of a PS to select between embryos produced by a couple with known family history of T1DM. If such a couple were to produce five embryos, the use of PGD and PS for T1DM could decrease their chance of having an affected child by 72%.

It is not only physical conditions which can be predicted using PS. Many psychological conditions have a significant genetic underpinning which can be quantified (to some extent) by these scores. The current most powerful PS for a psychiatric condition exists for schizophrenia, a debilitating condition characterised by recurrent episodes of psychosis.^9^ Schizophrenia is believed to have a heritability of 80%,^10^ meaning 80% of variance in population incidence is due to genetics. Given the ultimate limit to the power of PS is heritability, schizophrenia’s high heritability makes it a good candidate for reliable prediction. There are models that suggest a PS for schizophrenia could reach an AUC of 0.82.^11^ One 2019 study demonstrated a 4.6-fold increase in the odds of having schizophrenia in the highest PS decile compared with the lowest (although it is worth noting given the baseline is low, this still leads to a relatively low incidence of schizophrenia).^12^

Studies investigating the power of schizophrenia PS have uncovered another interesting finding: these scores are also associated with other psychiatric and medical disorders. Higher schizophrenia PS have been found to confer increased risk of anxiety, mood disorders, substance use, neurological disorders, personality disorders, suicidal behaviours and more.^12^ This phenomenon has been attributed to pleiotropy, the concept that genes can have multiple different effects on function, depending on the environment.

The purpose of these examples is not to imply we are currently at a stage where we can confidently apply PS to select an individual embryo. As outlined in the T1DM example, the accuracy of PS at a population level differs from accuracy at a family level. Family level PS is the relevant information for reproductive selection. Moreover, as with the schizophrenia example, an increased risk may still amount to an overall low risk rather than a high probability. The purpose is to show that this is a rapidly developing technology that is already in use in some jurisdictions (see the Regulation and ethics of PGD section), and is likely to develop increased accuracy at the family level that will likely produce increased interest in accessing this technology.

### Pleiotropy

This notion of pleiotropy is the driving factor behind one of the newest uses of PS. ‘Genomic indexing’ relies on risk for one condition correlating with risk for others. A series of papers with authors working at, or otherwise related to, the company Genomic Prediction argue and present data that ‘[s]everal achievements, including the ability to obtain accurate, genome-wide genotypes of the human embryo and the development of population-level biobanks, have now made PGT [preimplantation genetic testing] for polygenic disease risk applicable in clinical practice.’^13^ The idea is that through aggregating the relative PS for multiple conditions, weighted based on population prevalence and quality-adjusted life years, we can generate one singular score to correlate with predicted health.^13^ Someone with a lower genomic index score theoretically has a lower overall risk of developing a polygenic disease compared with someone with a higher score. When applied to a cohort of over 11 000 matched sibling pairs, a risk reduction was detected for all 11 conditions included in the genomic index score^i^ when one sibling was selected via genomic index testing compared with random selection.^14^ These relative risk reductions ranged from 4% for testicular cancer to 46.9% for heart attacks.^14^ (Genomic Prediction is listed as a conflict of interest on these papers as, per the Regulation and ethics of PGD section, the company is currently offering this testing.)

Pleiotropy has also linked apparently unrelated traits. For example, alongside increased risk for mental health disorders, schizophrenia PS have been linked to increased creativity.^15^ Choosing embryos with low risk of schizophrenia might inadvertently sacrifice embryos with a proclivity for the creative arts. Similarly, evaluations of PS for intelligence have demonstrated that these scores are positively correlated with autism risk.^16 17^

The ethics of PS is more than an extension of existing ethical issues around selection for monogenetic disorders. It raises issues of uncertainty (whether in fact the future child will develop the disease may depend on unknown environmental interactions), and the choice between suites of different risks and benefits. This paper does not address all of these issues.

### Regulation and ethics of PGD

PS are already in use. Just over 3 years ago, the company Genomic Prediction was formed. This New Jersey-based company currently offers PGD for both monogenic and polygenic conditions to consumers to ‘provide improved health to IVF families’.^18^ Their polygenic tests can be used to select against risk for a variety of child and adult-onset disorders including diabetes (types 1 and 2), schizophrenia, coronary artery disease, breast cancer, intellectual disability (ID), idiopathic short stature and more.^18^ Due to a liberal approach to regulation for assisted reproductive technologies (ARTs) in the USA, this technology can be accessed by any paying customer.^19^ Outside the USA, the more common regulatory model is disease-based, allowing selection against certain specified genetic diseases only. Disease-based models exist in various formats in Australia, the UK, Italy, Switzerland and France among others.^20 21^ As we shall see, many of these models are formulated in such a way as to be essentially inapplicable to testing for polygenic traits, (e.g., through requiring demonstration of genetic risk in order to access preimplantation testing) leading to uncertainty and potential inconsistencies between service providers.

### PS for non-disease traits

PS do not only provide probability estimates of developing common polygenic diseases, they can in principle provide probability estimates for continuous non-disease traits such as height, impulse control, personality type or intelligence where the prediction is not whether a specific disease will develop, but where individuals are projected to fall on a spectrum. The most data currently exist for predictions of intelligence because it is continuous with the disorder ID. We will focus on this as an example of non-disease trait but our arguments apply to all continuous non-disease traits.

Indeed, consumers can already access their personal genetic data relating to some non-disease traits. Gene-sequencing companies such as Geneplaza and DNA Land have recently added an intelligence PS to the list of genetic information they offer customers.^22^ Indeed, Genomic Prediction currently offers embryo testing for ID using this technology (though this test actually includes detecting predicted IQ in the low–normal range (it selects against predicted IQ below 75, while the current cut-off for ID is 70).^18 23 24^ The company currently would not screen for embryos predicted to have genetic potential for above-normal intelligence, however this is technically possible. Given the lack of PGD regulation in the USA, co-founder Stephen Hsu believes the public will demand this, and if his company does not offer it, another will.^25^

In the case of intelligence, uncovering significant genes has proved challenging, and thus the predictive power of PS is relatively weak.^2^ Intelligence is a highly polygenic trait—it is due to the cumulative (and possibly interactive) effects of thousands of genes and the environment. Twin and adoption studies have estimated that the heritability of intelligence is approximately 50%,^ii^ meaning the population variance in intelligence is 50% attributable to genetic differences.^26^ This 50% therefore sets the ceiling of predictive ability for PS.

Within that 50%, predictive ability remains challenging: with so many genes contributing to a single trait, each individual gene has a minuscule effect (possibly accounting for only approximately 0.005% of variance). Detecting these effects is possible by comparing genetic information and IQ scores of research participants, but it requires colossal sample sizes.

Moreover, there are difficulties in accurately assessing intelligence as distinct from education or test-training.^27^ Indeed, the most powerful PS have been developed from studies which use educational attainment (EA) (years of education completed) as a proxy for intelligence, as they are able to reach far greater sample sizes.^2^ These studies can predict both EA and general intelligence as measured by IQ scores, although their predictive ability is slightly better for EA. The current best intelligence predictor is a PS developed from a 2018 study involving 1.1 million participants.^28^ This score can account for 11%–13% of variance in EA, and 7%–10% of variance in intelligence measured as an IQ score.

It is postulated that as sample sizes continue to increase, the predictive ability of new PS will increase. However, there is an upper limit. The proportion of intelligence variance believed to be due to common genetic (single-nucleotide polymorphism) differences is 25%.^2^ To get from this 25% to accounting for the current estimate of 50% heritability of intelligence will require other methods, such as whole genome sequencing and investigating gene interactions.^2^ The feasibility of ever accounting for this ‘missing heritability’ has been questioned due to the complexity of gene-environment interactions and limitations of human research methods.^29^ Only time will tell what is ultimately possible in this realm.

Nevertheless, current PS already offer some probabilistic information. When participants from the 2018 study were split into PS quintiles, the disparity in college completion rate between top and bottom quintiles was around 40%.^28^ This PS is more predictive of college completion than household income. However, the lacunae in our understanding of the nature of the correlations remain problematic.^30^

For example, Peter Donnelly cautions: ‘Risk scores for IQ are very hard to interpret and hard to transfer between different countries and different ethnic groups. They’ll be capturing a lot of stuff that isn’t just fundamental biology, maybe genetic markers correlated with ethnicity or where people live.’^31^

Before PS can be used to predict intelligence, we will need not only correlation but plausible causal mechanism. We will consider other objections related to different forms of injustice presently.

Even if we did identify the genes that relate to intelligence rather than irrelevant correlates, these scores are inherently probabilistic. While they can provide reliable prediction when applied to populations, they are by no means deterministic for individuals. Some have labelled them ‘premature’^23^ and even ‘next to worthless’.^32^ Given the nature of intelligence and the complex factors which contribute to it, we will never be able to make a definite prediction based on genetics alone.

It is essential that further research determines the utility of PS for predicting non-disease traits because these traits will border with diseases traits, for example, the psychological trait of ‘psychoticism’ or hardheadedness blends with psychopathy, low intelligence blends into ID when IQ crosses from 71 to 69. If they are not validly predictive, they should not be used. But if they are predictive to a reasonable degree, the question arises: how do existing models of regulation in reproduction apply to them and how should they apply to them? We will not answer the last question but pose a novel model that has some advantages in dealing with PS in reproduction.

## Existing regulatory models

### Disease Model

Probably the most popular model of PGD regulation can be called the Disease Model. This model allows the use of PGD to select against embryos with disease traits, but not desired characteristics in what would be considered normal embryos. Countries which have adopted this model in one form or other include the UK, Australia, Italy, France and Switzerland among others.^20 21^ We will look at a couple of these in more detail as examples.

The UK’s approach to the model involves the generation of a comprehensive list of specific conditions approved for PGD use. This list is released by the Human Fertilisation and Embryology Authority (HFEA), and conditions are only included provided the authority is satisfied that embryo testing is carried out only where there is a ‘significant risk’ that a child born with the condition in question would have or could develop a ‘serious’ medical condition.’^33^ Tests involving PS do not currently appear on this list, and indeed will not unless the HFEA is satisfied that they measure a real and significant risk of developing a harmful condition.

A similar model exists in Australia, but in a different format. Instead of a list of permissible conditions, national guidelines recommend that PGD only be used to select against genetic conditions which ‘severely limit quality of life’.^21^ The guidelines must be followed to gain the accreditation required to offer ART services in Australia, and to receive government funding.^21^ It is left to service providers to judge which conditions meet the guideline criteria.

State legislation regarding PGD exists in Victoria, Western Australia and South Australia.^21^ The relevant acts differ in the specifics, but the result of each is very similar. Ultimately, access to PGD in these states requires medical demonstration that a couple is at risk of transmitting a genetic disorder.

This legislation is ill-suited for direct application to PS. For example, it is unclear whether the increased risk of developing a disorder *in certain environments* or *much later in life*, would be eligible. In the case of AUC, what level of uncertainty in our predictive ability can be tolerated within ‘significant risk’?

PS for continuous non-disease traits such as intelligence would not be allowed. However, there is provision to select against a number of conditions known to cause ID. If selecting against embryos with ID is an acceptable use of PS, then as polygenic tests become more widespread, accessible and reliable, it may be permissible under a Disease Model to use them to predict ID, provided they generate sufficient certainty of a ‘significant’ risk.

ID is currently defined as an IQ score of 70 or below, being 2 SDs below the mean of 100.^24^ Given this definition, ‘disease-based’ guidelines could be interpreted as permitting selection if IQ is expected to be 70 or below. However, it is not at all clear that this statistical distinction relates to a point of significant diminution in well-being or quality of life.^34^ Currently, disease-based models are not well adapted to testing for continuous traits, even though they do make provision for genetic disorders where the quality of life outcome is based on expected effects on those same traits.

A second development is increased use of IVF, leading to greater opportunity for both *primary* and *secondary* reproductive genetic testing.

Primary testing is the use of IVF and PGD for the purpose of selecting an embryo with a lower probability of disease. Initially this was limited to a handful of severe genetic disorders, like thalassaemia or Huntington disease, where prospective parents were known carriers. Given the burden on parents and the risk to the embryo, this was limited to the riskiest disorders. But the scope of IVF and PGD has significantly increased: 1 in 25 Australian births in 2018 were from IVF pregnancies,^35^ and in 2019, a form of preimplantation genetic testing was performed in 9.16% of IVF cycles.^36^ Furthermore, accessing whole genome or exome information has become significantly cheaper. Secondary testing is accessing genomic information secondary to PGD being performed for other reasons (eg, aneuploidy, single gene disorders, assessing embryo viability and so on). Such accessing of genomic information does not impose additional risks on the embryo as PGD is otherwise being performed in any case. It is not clear why secondary genomic testing for lower probability disease conditions should be banned.

For example, imagine IVF is being performed for infertility, including genomic testing of 10 embryos as part of the process in order to exclude catastrophic conditions. As it happens, they are all healthy. A genomic index could be derived to select the embryo with the lowest risk of common conditions.

In this way, testing for *low*-risk conditions as a part of at least secondary genetic testing could be justified. The alternative is applying exactly the same processes and risks, but picking an embryo at random.

Disease-based models have been effective in the past, as embryo testing was limited to known genetic disorders, in which a particular DNA pattern inevitably leads to significant disability and/or a high likelihood of suffering in the future child (such as Down syndrome or cystic fibrosis: although there are also some who argue that in fact these are also poor predictors of a low quality of life.^37^) The introduction of polygenic tests poses a further challenge to the disease-based model as it requires that we determine cut-offs for defining ‘significant risk’, ‘low quality of life’ and ‘disease’ in continuous traits such as intelligence.

### Libertarian Model

A more libertarian or laissez-faire approach to PGD exists in the USA, where there are no regulations concerning PGD, or funding for its use.^19^ Service providers can offer PGD at their discretion for any testable condition.^19^ Current professional guidelines simply recommend that decisions be left to clinicians, thus not providing further regulation.^19^ Consequently, PGD practice is governed by individual clinician decisions and market forces. Let’s call this the Libertarian Model.

The Libertarian Model allows selection for *any* disease or non-disease trait, regardless of its severity or the predictive power of the test. There would be no regulatory obstacle to Genomic Prediction offering PS for higher intelligence in embryo selection, and no barrier to clinicians using it either in primary or secondary testing.

Underlying this approach is philosophy such as Mill’s harm principle: ‘the only purpose for which power can be rightfully exercised over a member of a civilised community, against his will, is to prevent harm to others.’^38^ Yet classification of PGD as self-affecting or other-affecting is difficult.^39^ PGD certainly affects parents who adopt it, but does it affect their resultant children? As argued by one of us (JS), selection can only harm and wrong the individual selected if the condition resulting is so severe as to make life not worth living, because without this selection they would not have come to exist.^40^ This model allows the deliberate selection of embryos with genes linked to disabilities such as deafness, while the Disease Model forbids it (in the case of the UK, it is explicitly forbidden in legislation).

Nevertheless, there are several social concerns with the Libertarian Model:

- In the current American model, PGD is expensive (and in many other countries it is more readily available in private rather than public healthcare), and thus only a feasible option for the wealthy. This generates significant concerns about equality. As put by Peter Singer, ‘the present generation of wealthy people will have the opportunity to embed their advantages in the genes of their offspring’.^41^
- It is likely to reduce diversity and could be taken to imply lower value of those who have the selected-against traits.^42^
- This model gives undue power to individuals or companies in making significant reproductive decisions with no requirement for ethical input.
- Service providers are financially motivated, meaning ethical concerns may be disregarded in the face of monetary incentives.
- There is no regulation of service delivery and counselling, potentially putting consumers at risk of being subject to misinformation (particularly regarding the reliability of the test) and leading to harmful unrealistic parental expectations.

## The Welfarist Model

### What is the Welfarist Model?

We now propose a novel alternative model for the regulation of genomic testing in reproduction: the Welfarist Model. This model allows selection for any trait which is associated with well-being, or selection against a trait which reduces well-being. Well-being is broader than health or disease. Of course, what is a good life (or well-being) is much contested. It is beyond the scope of this paper to settle what constitutes well-being, though one of us has discussed it elsewhere.^34 43^ There are three major philosophical accounts of well-being: Hedonistic Theories, Desire Fulfilment Theories and Objective List Theories.^44^

According to the Welfarist Model, the use of PS should be allowed if they are correlated with a greater chance of a life with more well-being. This requires a philosophical and ethical analysis of what constitutes well-being and a scientific analysis of the correlation between a PS and that outcome.

There are two versions of such a model. A scalar version would allow any test which reliably tracked a greater chance of greater well-being. A threshold version would allow testing if there were a reliable sufficient increase in expected welfare, where expected welfare is the probability of a welfare enhancement multiplied by the magnitude of that enhancement. A threshold version is closer to the current Disease Model and is more likely to be socially acceptable. We will assume a threshold version.

The Welfarist Model can more easily accommodate polygenic risk scores for disease: if the probability is sufficiently high, then the test could be used. The bar could be set lower than at present: for example, a statistically significant increase might be sufficient. Issues of pleiotropy would need to be addressed where this is an issue.

A Welfarist Model also has greater capacity to address non-disease states or continuous variables such as intelligence. What matters is whether the non-disease state is correlated or not with well-being. In this way, the Welfare Model would be similar to the Disease Model in that it would allow for selection against low IQ up to a certain threshold, however it differs in the way this threshold is determined. While the current cut-off is purely statistical in the Disease Model, the Welfarist Model instead defines a cut-off based on expected well-being.

To illustrate using the example of PS for intelligence, the current definition of ID uses a statistical concept of normality to define ‘disability’ (IQ <70). There is some evidence that individuals who fall in the low–normal range (70–85) still struggle with many elements of daily life, but are often ineligible for supportive resources without a diagnosis of ID.^45^ The Welfarist Model could allow polygenic testing to predict IQ less than 85, rather than 70, if the obstacles to well-being of having low–normal IQ were significant enough.

### Does intelligence relate to well-being?

This question would have to be answered before polygenic testing for low–normal IQ would be permitted on the Welfarist Model. There is little value in intelligence if all it translates to is a higher score on an IQ test. There is some evidence that IQ level is positively correlated with socioeconomic status, income, EA, health and even decreased mortality rate by middle age, which may be seen as objective measures of a good life.^28 46–48^ An IQ of 75–80 has been identified as the current threshold for employability, meaning even those in the low–normal IQ range (IQ of 70–85) face significant difficulties.^49^ Studies assessing subjective well-being have also found that people with higher IQ levels tend to rate their lives as being happier than their less intelligent counterparts.^50^ However, these results are contested and were highly mediated by income, health and independence among other factors. IQ seems to largely influence happiness through its impact on these other factors, rather than providing a direct cause: therefore, social change to divorce these factors from intelligence would resolve the issue. Such issues would need to be resolved by further research. On a Welfarist Model, if well-being is *not* inextricably linked to intelligence (i.e., if it is only linked through changeable social structures), then it would no longer be a relevant part of reproductive selection.

### Generating a threshold?

It may be simple to say that certain genetic disorders impact welfare, but it is far more challenging to set a threshold of acceptable well-being for continuous traits such as intelligence.

While there may be a positive correlation between intelligence and well-being, this link seems to exist primarily through the impact of IQ on other life outcomes such as health, income, occupation and socioeconomic status. Of course, the link between the outcome and IQ is mediated by social structures and policies that can enhance such inequalities in outcomes or minimise them, and there is therefore reason to believe that such differences could be reduced. However, these outcomes present a starting point for determining a well-being-based IQ cut-off. Many of these factors are themselves continuous, meaning drawing a line of ‘well-being’ which is not arbitrary or statistical is fraught. In research into the genetics of intelligence, researchers have used a proxy which is itself of value: high school completion. The prediction of such an outcome could be used if there were a robust genetic contribution in addition to social contribution. Education is recognised as a fundamental human right under the United Nations’ universal declaration of human rights, highlighting its importance for well-being.^51^ A PS linked to high school completion—although still arbitrary—at least has some relevance for well-being rather than simply a statistical deviation from the norm. Completing secondary school increases opportunities through facilitating higher education and access to fulfilling jobs. Those who drop out are more likely to be unemployed, live in poverty, commit crimes and even die earlier.^52^ A 2007 study demonstrated that ‘education is one of the strongest predictors of health’.^53^ The authors even proposed that high school dropout should be recognised as a public health issue.

The current most powerful PS can predict 7%–10% of intelligence variance, but 11%–13% of variance in EA.^28^ This PS is better at predicting EA than intelligence as measured by IQ scores. If we sought to generate a PS threshold (rather than a direct IQ threshold), there would be a significant benefit to using EA—we could adopt the PS correlating with the EA outcome (secondary school completion) as a threshold, which would increase the reliability of the test.

There is one significant caveat. As mentioned above, one possible reason for the discrepancy is that PS identifies genetic correlates that are unrelated to intelligence. These may be completely unrelated, or it could indicate that it selects for other non-intelligence factors which aid in advancing education.iii Plausible factors include conscientiousness or interest in academia. Selection of irrelevant traits is clearly a catastrophic failure, but selection for other relevant traits may be considered a drawback or a strength of the score, depending on whether or not it is desirable in terms of well-being to select for these factors. As stated above, significant advances in our current understanding would be needed to exclude catastrophic potential failures such as this which would render the scores useless.

### Funding options

One factor which must be considered in proposing the Welfarist Model is funding. There is much concern that if we allow any form of selection for intelligence it will be only accessible to the wealthy, thus compounding their cognitive and economic advantages over others. The provision of universal funding for the Welfarist Model may be the only way to entirely elude social justice concerns, however such a model raises questions about opportunity cost. If this scheme will lead to greater suffering in other sectors without providing overwhelming benefits, it cannot be justified. Accordingly, public funding for PGD in line with the Welfare Model will only be defensible if:

1. **It is cost-effective**: to justify the use of public money, funding for the test should be a cost-effective measure. This includes first that the test will be reliable (provide meaningful prediction) and second that there will be an adequate prevalence of low PS for the test to detect—if the test rarely identifies embryos with a score below the threshold level, it is unlikely to be cost-effective. Finally, it will require that this is the best use of these funds and that other measures are not more effective at increasing well-being.
2. **It is generalisable:** the test in its current state is most effective in populations of European ancestry due to the databases used in its development. When applied to other populations, its predictive value is significantly diminished.^28^ As such, benefits from the test may be disproportionately high in European populations. Given the current nature of society in which people of European ancestry tend to face far fewer barriers than those of other races, it would be wrong to further their advantage by funding a test which accords them additional benefits over others.
3. **It provides welfare-relevant benefits:** benefits associated with creating children with enough opportunity for a flourishing life (well-being) are undeniable, but the weight of these benefits may be questioned due to their impersonal nature.^iv^

There would clearly need to be significant progress in PS technology before this could be considered. In addition, it is unlikely to be cost-effective to offer IVF and PGD solely for the purpose of polygenic testing. However, it may be cost-effective to add such testing for couples otherwise undergoing IVF and PGD, that is, secondary testing. The cost-effectiveness of funding PGD against known genetic disorders is well-established.^54^ For couples already undergoing PGD, the additional cost of providing the polygenic test would not be excessive. What is more, after selecting out those with genetic disorders, couples are likely to be left with multiple embryos to select between for implantation. In such situations, since a choice must be made between embryos, using welfare-relevant information derived from PS appears better than purely relying on chance.

## Objections to the Welfarist Model

The Welfarist Model provides a regulatory framework which overcomes some concerns with existing models. On top of creating a more meaningful threshold for selection, it is applicable to both discrete (eg, single gene disorders) and polygenic conditions, it provides improved clarity regarding the permissibility of testing and offers access to the benefits of selection for intelligence. However, it also raises a number of foreseeable objections.

### Unreliability

One major concern about this technology in relation to the example of predicting intelligence is that current PS can only account for 10% of variance.^28^ That may be an overestimate. Those with low PS can achieve highly, while those with high scores would not necessarily be smarter than average.^23^ While the technology is not yet mature, it may not need to be unrealistically perfect. Provided prospective parents are well informed about its limitations (which is, admittedly, a big ‘if’), a test that can identify a significant risk of a score that predicts a low well-being outcome would be equivalent to some disorder probabilistically identified by the Disease Model. Indeed, the problem of unreliability applies equally to the Disease and Libertarian Models.

The important question is not the current value of the technology: it is clearly not currently useful. Given recent rapid progress in this field it is likely that PS reliability will continue to increase substantially in the future. We ought to consider how these tests might be regulated so that there is a well thought-out framework in place.

### Eugenics

‘Eugenics’ literally means ‘well born’ and applies to a wide range of practices aimed at influencing the genetic make-up of the next generation. It is typically understood today in a pejorative sense to be the intentional attempt to bring about a healthier or ‘better’ population,^55^ especially by coercion or limiting options available, especially when there is state involvement^56^ in this by coercion or limitation of options.^57^

There have been two major evils brought about through policies based on eugenic principles: first, an implication that there is less value in the lives of individuals of lower health or well-being; and second, the involuntary imposition of such policies on reproductive decision-making. Eugenics has been used to justify horrific acts. In the early 1900s, America, Scandinavia, Nazi Germany and other European countries pursued eugenic goals in various ways, including the murder and involuntary sterilisation of those deemed ‘unfit to reproduce’ due to low IQ, disability, mental illness, alcoholism, unemployment, criminality, race, religion or poverty.^57^ This ideology culminated in the holocaust, in which the Nazis murdered millions of Jewish people and other groups they considered to be ‘unworthy of life’.^58^

Modern clinical genetics (and the current Disease Model) aims at using genetic knowledge to enable couples to have the free choice to select an embryo free of genetic disorders that have a significant effect on the predicted quality of life of the resulting child. In the broad general sense, it is eugenic but it is not eugenic in the pejorative sense of aiming at ‘improving’ the population, especially through the use of coercion. In this sense, the Welfarist approach is merely an extension of the Disease Model. It is eugenic in the same general sense as the current Disease Model, because it results in selection based on genetic information, but not in the pejorative sense. There is no broad social goal or coercion employed.

Health and well-being are reasonable goals for society to aim for in terms of its general policies—there are many health and education measures in place aiming to improve the health and prospects of children. Currently, society does support policies that do allow parents to select against certain conditions. For example, PGD to select against genetic conditions which contribute to ID, such as Down syndrome or Fragile X syndrome, are common and are even publicly funded, implying not only assent, but active support for allowing prospective parents to select against these conditions. Selection on the basis of a PS, if it is well correlated and causally linked to a welfare threshold with important bearing on the future child’s well-being is ethically equivalent to these. Indeed, allowing selection on the basis of only *some* genetic conditions may be discriminatory.

It would be consistent with an anti-eugenic stance to reject *all* forms of selection. But if we accept a Disease Model, then a Welfarist Model is not more eugenic. Instead, it focuses attention on factors that are likely to affect the child’s well-being, instead of on certain genes that have been designated as disease-causing. Indeed, the objection from eugenics applies equally to the Disease Model and the Libertarian Model (sometimes called ‘Liberal Eugenics’).

As with the current Disease and Libertarian Models, there should be no governmental interference in reproductive decision-making. Prospective parents should be free to adopt or reject the technology as they wish, just as they can in the Disease or Libertarian Models. This is in line with modern genetics which focuses on providing information and opportunity, eschewing coercion. The only difference in moving to a Welfarist Model from the Disease Model is the focus on well-being, rather than statistical deviation from the mean. This is consistent with the WHO definition of health as ‘complete physical, mental and social well-being.’^59^

### Parent–child relationships

There are fears that allowing parents to select children based on genetic traits will negatively impact parent–child relationships. Eminent critic Michael Sandel is afraid that this technology ‘threatens to banish our appreciation of life as a gift’, and that it portrays an attitude towards children which is not conducive to good parenting.^60^ This concern may in fact be more fitting in cases of gene editing, rather than genetic selection. Children born following selection decisions still have a unique and uncontrolled set of genes. This pattern of DNA, be it due to chance, fate, God or something else, is free from human influence. Parents have chosen to bring this particular child into existence, however, whichever genetic traits they happen to possess have not been altered. In this way selection is importantly different from gene editing, in which parents alter pre-existing genetics. In the case of genetic modification, a child could rightfully complain about the enhancement; in the case of selection, they could not.

A further concern is that of ‘hyperparenting’. Hyperparenting occurs when parents limit a child’s freedom due to a desire for them to achieve, for example, through obliging them to study, practise music or train for sports excessively against their will. There is little doubt that hyperparenting can violate a child’s autonomy and impede well-being, however there is no necessary or inevitable link between genetic selection and hyperparenting. Hyperparenting is bad parenting and it occurs without polygenic testing. While parents with a tendency to hyperparent may be likely to be interested in ‘designer children’, these parents are likely to hyperparent regardless of the method of procreation.^61^ Hyperparenting certainly restricts autonomy, and is something we ought to eradicate, but allowing selection using PS does not undermine autonomy.

### Entrenches unjust discrimination

The Welfarist Model and the principles behind it have the potential to be extended to other traits. While many of these—such as impulse control, concentration ability and memory—may be appropriate, an emphasis on welfare rather than ‘normality’ or ‘health’ may lead to more harmful applications. Notably, factors such as race, sex and gender could be said to diminish expected well-being due to prejudice and discrimination.^31^ A repugnant implication of the Welfarist approach could therefore be to support selection against these characteristics.

Employment of PS for intelligence or EA may entrench discrimination in another important way. Much of the disadvantage and adverse effect on well-being of having a lower IQ is due to structural injustice and the way society’s institutions are organised. In addition, there is a large and important literature on how IQ testing, and the project of finding genetic contribution to intelligence, can never be ‘socially neutral’, but instead supports the status quo, and instead of being separable from its racist and sexist history and influences, has the goal of legitimising social hierarchies built into it.^62^ Moreover, science is never separate from society. Ensuring association between PS and IQ or EA is not the result of structural injustice, rather than genes may not be achievable, given the nature of PS research and the inability to extract subjects from the pervasive environment. By using such scores, we may be perpetuating this fallacy and injustice.

In short, some argue that rather than looking for associations between genes and intelligence, or using PS in reproduction, we should be correcting structural injustice and the social/environmental determinants of disease and disadvantage. That will better promote well-being.

These concerns are clearly valid. We should be aiming to correct social injustice. However, biology and genetics do play some role in the development of our individual traits, and these traits have some bearing on how well our lives go under almost any social conditions. The project of understanding this role, although pursued with greater humility and self-awareness, is not in opposition to social change.

Moreover, such objections apply equally well to the existing Disease and Libertarian Models, or indeed are more adequately addressed by a Welfarist Model. For example, much disease is the result of social injustice, not genetic disposition. It may equally be objected that any disadvantage that accrues to being intellectually disabled (IQ <70) is the result of injustice and the way society is organised. Indeed, some people with and parents of children with Down syndrome (for example) argue that the lives of those with Down syndrome afford equivalent well-being to those without ID. On the Welfarist Model, this argument would mean there would be no reason to screen to select against Down syndrome, but it would not change the status quo on the Disease Model. On a Libertarian Model, parents are free to select against or in favour of disability, according to their own beliefs.

Insofar as objections from social injustice do apply to the Welfarist Model, they will also apply to the Disease Model. The Libertarian Model is more immune to this objection because parents can select in favour of disease or disability. However, given that in the current prevailing culture, most parents are likely to select against these, in practice the Libertarian Model is likely to perpetuate injustice.

The Welfare Model should only be applied to select against characteristics which are likely to lead to a below threshold level of well-being, even under conditions of justice.^34^ In the absence of this proviso, untrammelled application of the model could generate significant harms by reducing diversity and denormalising traits which are the basis of unwarranted discrimination, and which are appropriately addressed by social means (combating prejudice and discrimination), or by perpetuating social injustice. Nevertheless, diversity *itself* is not intrinsically valuable. Peter Singer highlighted this with the example of life expectancy, for which greater diversity requires more people dying younger.^41^ However, we have much to lose by eliminating diversity in race, sex, sexuality and other traits that are only connected to well-being through injustice.

### Why limit enhancement?

If we accept that reproductive selection for cognitive ability is permissible, then why not disregard the threshold entirely and select the embryo with the highest PS? Why not move from the threshold to the scalar version of the Welfarist Model?

The Welfarist Model is intended to address the ‘arbitrary line’ problem of the current disease-based model. Where traits are continuous, it replaces a standard deviation-based concept of ‘disease’ and ‘normality’ with one of ‘disadvantage’. Welfare is a better standard because it addresses what matters to the future child. Nevertheless, welfare is necessarily broad. It is dependent on environment and on a range of factors, with cognitive ability only being one. It remains unknowable what exact range of genetic attributes, environment and experiences lead to the greatest welfare. For that reason, addressing only a range where there is strong evidence that a score is highly likely to directly lead to a significantly lower welfare outcome is proposed.

### Expressivist concerns

The ‘expressivist critique’ is one of the most common arguments against the use of PGD in any setting, including against the Disease Model. The argument holds that through selecting only non-disabled (and in this case non-disadvantaged) embryos for implantation, we are expressing a view that disabled people ‘should not have been born’ and are less valuable than non-disabled people.^42^ This objection applies equally to the existing Disease Model as it does to the Welfarist Model.

One way to counter the expressivist critique is to consider PGD as a preventative health measure.^63^ It seems illogical to say that preventing an illness (e.g., by vaccination) is disrespectful to its sufferers. We do not consider it impermissible for women to take folic acid during pregnancy to prevent neural tube defects; in fact, this is strongly encouraged. This does not mean that people with neural tube defects or vaccine-preventable illnesses are of lesser value. The moral value of a life is distinct from the quality of that life.^61^

In cases of selection however, we necessarily conflate the disability with the embryo, as we cannot (yet) select the embryo without also selecting its disability. This still does not make selection wrong. For one thing, selecting one embryo over another cannot be seen to harm either embryo or future child (unless we are to select an embryo which has a genetic disorder that will lead to the future child having such poor quality of life that their life is not worth living). The embryo who is not selected never comes to existence as a person, and therefore cannot claim to have been harmed. Moreover, embryos are not people. Presumably then, the relevant harms here are experienced by existing people, who are affected by the act of discrimination against future people with their disabilities. However, how we value and treat people is independent of how we treat embryos. Embryos must be destroyed by law in some Australian states (eg, Victoria)—people must not be killed. Parents adopting PGD are not doing so because they believe disabled people lack moral value, any more than parents seeking to avoid asthma in their children are expressing a negative view of asthmatics. It is far more likely that they are simply acting to secure what they see as the best possible life for their future child.^42^ In the end, parents should (and generally do) love and accept the child they have, whatever genetics or fortune the child experiences.

Finally, this model does not mandate that parents adopt this, or any other, genetic test to select their children. Couples are free to reject the technology, or ignore information gleaned from the test when making reproductive decisions. Through permitting the use of PGD for genetic disorders, we have already accepted that parents have the right to select against genetic traits in their offspring. Extending the model from health to well-being, to select against disadvantage as well as disease, is a reasonable step.

## Conclusion

PS may possibly revolutionise genomic selection in reproduction. Regulation developed 40 years ago needs to evolve. We should consider moving from a Disease to Welfarist Model. A Welfarist Model is better placed to deal with the probabilistic nature of information provided by PS and is able to address the issue of testing for polygenic contributions that lead to spectrum rather than binary outcomes.

The introduction of a Welfarist Model requires significant further work. It requires settling on a conception of well-being. But this surely is among the most urgent tasks for society and individuals. It is what should guide our education, social welfare and legal institutions. As for individuals, it is what constitutes a good life. These tasks are philosophical and ethical.

It also requires more scientific research to establish the genetic contribution to well-being under conditions of social justice. This requires not merely statistical correlation but understanding causal mechanism to avoid irrelevant correlations. It requires careful consideration of the extent to which injustice and other differences in environment play a role in outcomes.

The Welfarist Model offers a regulatory framework which addresses major concerns generated by existing models. It is ethically justifiable, feasible, applicable to polygenic tests, and even seems to reflect principles underlying current regulation. Additionally, the use of an outcome-based threshold rather than a statistical one ensures wide normative benefits.

Nevertheless, the Welfarist Model is one model among several. What is most important is that we give adequate thought to the regulation of this new technology—underestimating its importance could prove catastrophic. Further research into the reliability of PS, likely future impacts of selection for non-disease states, and public opinion on various policies would be valuable in progressing thought and action in this area. We may only have one chance to shape the impact of novel technologies; we must seize this opportunity and work towards improving lives and achieving a fair and just society.

## Footnotes

Basal cell carcinoma, breast cancer, malignant melanoma, prostate cancer, testicular cancer, coronary artery disease, heart attack, hypercholesterolaemia, hypertension, type 1 diabetes and type 2 diabetes.

In fact, the heritability of intelligence increases as we age, from around 20% in infancy to as high as 80% in late adulthood. This is believed to be due to ‘genetic amplification’ according to which children (and adults) modify and select their environments in line with their genetic propensities.^64^

This is inherent to the score itself (due to its method of development), and not only when applied to predicted educational attainment.

Selected children do not personally benefit from increased intelligence, as it is a condition of their existence. Benefits are impersonal in the sense that they impact the world as a whole, rather than individuals. Presumably, a world with less suffering is an inherently better world even if no individuals are personally impacted. This concept was first described by Derek Parfit, and is called the ‘non-identity problem’.^65^
